# Effects of cooking techniques on fatty acid and oxylipin content of farmed rainbow trout (*Oncorhynchus mykiss*)

**DOI:** 10.1002/fsn3.512

**Published:** 2017-08-19

**Authors:** Katrina Flaskerud, Michael Bukowski, Mikhail Golovko, LuAnn Johnson, Stephen Brose, Ashrifa Ali, Beth Cleveland, Matthew Picklo, Susan Raatz

**Affiliations:** ^1^ Department of Food Science and Nutrition University of Minnesota St. Paul MN USA; ^2^ USDA ARS Human Nutrition Research Center Grand Forks ND USA; ^3^ Department of Biomedical Sciences University of North Dakota School of Medicine and Health Sciences Grand Forks ND USA; ^4^ USDA Agricultural Research Service National Center for Cool and Cold Water Aquaculture Kearneysville WV USA

**Keywords:** cooking technique, farmed rainbow trout, fatty acids, oxylipins

## Abstract

The aim of this study was to investigate the effect of various cooking techniques on the fatty acid and oxylipin content of farmed rainbow trout. Rainbow trout is an excellent source of long‐chain omega‐3 (*n*‐3) polyunsaturated fatty acids (PUFA) which have beneficial health effects. Fillets of 2‐year‐old farmed rainbow trout were baked, broiled, microwaved, or pan‐fried in corn (CO), canola (CaO), peanut (PO), or high oleic sunflower oil (HOSO). Fatty acids and oxidized lipids were extracted from these samples and their respective raw fillet samples. Fatty acid content was determined using gas chromatography and oxylipin content by mass spectroscopy. The values obtained from each cooking method were compared to those obtained from the respective raw fillets using paired *t* tests. PUFA content was not altered when samples were baked, broiled, microwaved, or pan‐fried in CO or CaO. Pan‐frying in PO reduced α‐linolenic acid (18:3*n*‐3), eicosadienoic acid (20:2*n*‐6), and dihomo‐γ‐linolenic acid (20:3*n*‐6), while pan‐frying in HOSO reduced 18:3*n*‐3, eicosapentaenoic acid (20:5*n*‐3), docosapentaenoic acid (22:5*n*‐3), docosahexaenoic acid (22:6*n*‐3), linoleic acid (18:2*n*‐6), 18:3*n*‐6, 20:2*n*‐6, 20:3*n*‐6, docosatrienoic acid (22:2*n*‐6), and adrenic acid (22:4*n*‐6) compared to raw fish. Cooking decreased the omega‐6 (*n*‐6) PUFA‐derived oxylipins, but caused no change in 20:5*n*‐3 or 22:6*n*‐3‐derived oxylipins of the fillets. In conclusion, pan‐frying was the only cooking method to alter the fatty acid content of the fillets, while observed changes in oxylipin content varied by cooking method. As the physiological impact of oxylipins is currently unknown, these results suggest that the cooking methods which optimize the consumption of *n*‐3 PUFA from rainbow trout are baking, broiling, microwaving, or pan‐frying in CO, CaO, or PO.

## 1 INTRODUCTION

Studies show that a decreased risk of cardiovascular disease (CVD) is correlated with a diet high in long‐chain (LC) omega‐3 (*n* **‐**3) polyunsaturated fatty acids (PUFA) (Bang, Dyerberg, & Nielsen, 1971; Bang, Dyerberg, & Sinclair, 1980; Mori, 2014; Psota, Gebauer, & Kris‐Etherton, 2006; Virtanen, Mozaffarian, Chiuve, & Rimm, 2008). The primary dietary source of LC*n* **‐**3 PUFA is fatty fish (Mori, 2014). PUFAs play an important role in inflammation reduction. The LC*n* **‐**3 PUFAs eicosapentaenoic acid (EPA; 20:5*n* **‐**3) and docosahexaenoic acid (DHA; 22:6*n* **‐**3) may assist in this role by inhibiting the formation of proinflammatory eicosanoids and cytokines (Calder, 2015; Gabbs, Leng, Devassy, Monirujjaman, & Aukema, 2015; Tjonahen et al., 2006).

A variety of methods of cooking fish have been evaluated for their ability to change fatty acid content and induce changes in fatty acid metabolites. The preparations include baking, boiling, deep‐frying, grilling, microwaving, pan‐frying, roasting, and steaming. Overall, the data indicate that there is an effect on the composition of various fatty acids, with changes in LC*n*‐3 PUFA depending on the cooking method and the variety of fish (Agren & Hanninen, 1993; Al‐Saghir et al., 2004; Ansorena, Guembe, Mendizabal, & Astiasaran, 2010; Asghari, Zeynali, & Sahari, 2013; Echarte, Zulet, & Astiasaran, 2001; Sioen et al., 2006; Tokur, 2007; Uran & Gokoglu, 2014).

Oxylipins can be used as a measurement of lipid oxidation, indicating which fatty acids are most impacted by heat. Oxylipins are oxidized products of PUFA (Gabbs et al., 2015). Omega‐6 (*n*‐6)‐derived oxylipins tend to have increased inflammatory and vasoconstrictive effects, while *n*‐3‐derived oxylipins tend to be anti‐inflammatory (Calder, 2015; Gabbs et al., 2015; Ray et al., 2015). It is unknown how the method of preparation affects the oxylipin content of fish fillets and the potential health impact of those oxylipins. Answering these questions will determine if there is a superior method of preparation that maintains the beneficial lipids found in fish.

Farmed rainbow trout contain a high content of LC*n*‐3 PUFA relative to other common food fish species such as tilapia and catfish (Weaver et al., 2008). Research on the impact of various cooking methods has been conducted on rainbow trout (Agren & Hanninen, 1993; Asghari et al., 2013; Gokoglu, Yerlikaya, & Cengiz, 2004; Tokur, 2007). However, the effect of pan‐frying with various oils, microwaving, baking, and broiling on both the fatty acid and oxylipin profile of rainbow trout has not been investigated. Therefore, the aim of this work was to determine the effects cooking methods have on both the fatty acid and oxylipin content of farmed rainbow trout.

## 2 MATERIAL AND METHODS

### 2.1 Preparation of fillets

Farmed rainbow trout (*Oncorhynchus mykiss*) were hatched and reared at the USDA, ARS, National Center for Cool and Cold Water Aquaculture in Kearneysville, WV. The fish were raised in partially recirculated water (12–13°C) in 3 m^3^ tanks and fed daily with a commercially available diet (Finfish G, Zeigler Bros, Inc., Gardners, PA) through automatic feeders. The feed was dispensed at or just below satiation levels and adjusted over time. After 2 years the trout were harvested in water containing a lethal dose of tricaine methanesulfonate (300 mg/L) (Western Chemical, Ferndale Washington). The fish were hand filleted immediately upon death and fillets were stored at −80°C until they were shipped on dry ice to the USDA, ARS Grand Forks Human Nutrition Research Center, Grand Forks, ND. Once the fillets were received, they were stored at −80°C until processing.

Fish fillets were moved from the −80°C freezer to a −20°C freezer. After 3 days, the fillets were moved from the −20°C freezer into a 4°C refrigerator. The following day the fillets were removed from the refrigerator and were prepared. Each fillet was weighed and then cut into 55 g pieces longitudinally (dorsal–ventral) along the fillet. A 5 g piece, to serve as the raw sample, was cut from each of the 55 g fillet samples (from either the dorsal or ventral side), stored in a zip‐lock bag, and placed into the −80°C freezer until processing. The remaining 50 g pieces were then stored in the 4°C refrigerator until cooked.

### 2.2 Cooking of fish

Cooking techniques were based on previously published methods (Al‐Saghir et al., 2004; Asghari et al., 2013; Raatz et al., 2011). For each cooking method, samples were cooked in triplicate. As soon as the desired temperature was attained by all cooking methods, the samples were cooled and frozen at −80°C until analyzed.

#### 2.2.1 Pan‐frying

Eleven grams of test oil: peanut oil (PO) (Planters, Kraft Foods North America, East Handover, NJ), high oleic sunflower oil (HOSO) (Cargill Food Innovation Center, Minneapolis, MN), corn oil (CO) (ACH Food Company, Inc., Memphis, TN), or canola oil (CaO) (Cargill Food Innovation Center, Minneapolis, MN) were added to the center of a 10.5″ (26.7 cm) electric frying pan (National Presto Industries, Inc., Eau Claire, WI). The frying pan was set to 177°C (350°F). When this temperature was reached, the 50 g sample, with a thermometer placed at the center of the fillet, was added to the pan in the center of the oil. The fillet was flipped over to the other side midway through cooking and cooked until the core temperature attained 63°C (145°F) for 15 s (The National Restaurant Association, 2010).

#### 2.2.2 Baking

A conventional oven was preheated to 177°C (350°F). A 50 g sample, with a thermometer placed at the center of the fillet, was placed in a 5.5″ × 4.5″ (14 cm × 11.4 cm) bake and serve container (Pactive Pressware, Columbus, OH) and baked until the core temperature reached 63°C (145°F) for 15 s (The National Restaurant Association, 2010).

#### 2.2.3 Oven broiling

An oven rack was placed 4.5″ (11.4 cm) away from the heating source. The oven was then preheated to 260°C (500°F). A 50 g sample, with a thermometer placed at the center of the fillet, was placed in an 8″ × 8″ (20 cm × 20 cm) Pyrex glass container which had 3 g of CO spread on the bottom. The sample was placed into the oven, 4.5″ (11.4 cm) away from the heat source, and was cooked until the core temperature reached 63°C (145°F) for 15 s (The National Restaurant Association, 2010). The fillet was flipped over to the other side midway through cooking.

#### 2.2.4 Microwaving

A 50 g sample was placed in a 5.5″ × 4.5″ (14 cm × 11.4 cm) bake and serve container (Pactiv Pressware, Columbus, OH). Wax paper was used to cover the container. The sample was then placed into a 1,200‐W high‐power microwave (Panasonic, lot # NN‐SA661S). The sample was cooked in short intervals until the core temperature reached 63°C (145°F) for 15 s (The National Restaurant Association, 2010).

### 2.3 Total fatty acid analysis

#### 2.3.1 Lipid extraction

Frozen fillet samples were pulverized in liquid nitrogen. Lipids were extracted using a modified Folch method (Folch, Lees, & Sloane Stanley, 1957). Briefly, 50 mg of pulverized fish were weighed into a 6‐ml test tube, combined with 2.5 ml of chloroform (Sigma‐Aldrich, St. Louis, MO) : methanol (J. T. Baker, Austin, TX) (2:1 v/v) + 50 μmol/L 2,6‐di‐tert‐butyl‐4‐methylphenol (BHT) (Sigma‐Aldrich, St. Louis, MO) and 0.5 ml of 0.9% KCl (Sigma‐Aldrich, St. Louis, MO) + diethylenetriaminepentaacetic acid (DTPA) (Sigma‐Aldrich, St. Louis, MO), vortexed, and then mixed overnight on a rotating test tube mixer at ambient temperature. Subsequently samples were centrifuged and the organic layer was removed. The aqueous layer was extracted with a second 2.5 ml aliquot of chloroform:methanol (2:1 v/v) + 50 μmol/L BHT, which was combined with the first organic layer. Samples were dried under nitrogen and stored at −20°C until analysis.

#### 2.3.2 Fatty acid methyl ester analysis

Extracts were redissolved in 1 ml of hexane (Sigma‐Aldrich, St. Louis, MO) : isopropanol (J. T. Baker, Austin, TX) (3:2 v/v) + 50 μmol/L BHT; 100 μl was transferred to a clean 6 ml test tube and dried under nitrogen. Nonadecanoic acid (Nu‐check Prep, Inc., Elysian, MN) was added as an internal standard (50 μl of 3 mg/ml in methanol) along with 2 ml of anhydrous methanol (Sigma‐Aldrich, St. Louis, MO) : acetyl chloride (Sigma‐Aldrich, St. Louis, MO) (19:1 v/v). Samples were incubated overnight at 25°C. The transesterification reagent was quenched with the addition of 0.5 ml 1.4 mol/L potassium carbonate (Sigma‐Aldrich, St. Louis, MO), and extracted into 2 ml hexane. The hexane layer was removed, dried under nitrogen, and reconstituted in 250 μl of hexane for GC‐FID analysis. Analysis was performed on a Thermo Trace‐1310 gas chromatograph (Thermo Scientific, Waltham, MA) equipped with a Supelco SP‐2560 capillary column (75 m, 0.18 mm i.d., 0.14 μm film thickness, Sigma‐Aldrich, St. Louis, MO). The samples were injected with a split of 100:1 at 250°C with a column flow rate of 2 ml/min. The profile used for the oven temperature was adapted from Massod et al.: 150°C held for 0.25 min, increased to 200°C at a rate of 35°C/min, then increased to 225°C at a rate of 8°C/min, and held at 225°C for 4 min once the temperature was reached (Masood, Stark, & Salem, 2005). The acquired data were analyzed using a Dionex Chromeleon 7.2 Chromatography data system (Thermo Fisher Scientific, Waltham, MA). Analysis of the oils used for pan‐frying followed the same gas chromatography method. Table 1 presents the analyzed fatty acid content of the oils used.

**Table 1:** Analyzed fatty acid content of oils used in pan‐frying

Fatty acid	PO	CO	CaO	HOSO
14:0	0	0	0	0.11
14:1*n*‐5	0	0	0	0
16:0	10.11	10.94	3.53	3.83
16:1	0	0	0.21	0.14
18:0	2.39	1.73	3.11	4.48
18:1*n*‐9	58.66	23.92	65.06	82.26
18:1*n*‐7	0	2.80	3.42	1.06
18:2*n*‐6	23.85	54.98	20.56	5.88
20:0	1.85	1.17	0.70	0.29
18:3*n*‐6	0	0	0.36	0
20:1*n*‐9	0	0	0	0
18:3*n*‐3	0	1.71	2.30	0.12
22:0	3.15	0	0.51	1.06
20:3*n*‐6	0	0	0	0
20:5*n*‐3	0	0	0	0
22:5*n*‐3	0	0	0	0
22:6*n*‐3	0	0	0	0
Total SFA	17.50	13.84	7.85	9.77
Total MUFA	58.66	26.72	68.69	83.46
Total PUFA	23.85	56.69	23.22	6.00
Total *n*‐6	23.85	54.98	20.92	5.88
Total *n*‐3	0	1.71	2.30	0.12
Total	100	97.25	99.76	99.23

Data expressed as g/100 g; *n* = 1.

### 2.4 Lipid oxidation product analysis

#### 2.4.1 Extraction of lipid oxidation products

Oxidized lipids were extracted from tissue as described previously (Brose, Thuen, & Golovko, 2011; Golovko & Murphy, 2008; Raatz et al., 2011). Briefly, tissue was pulverized under liquid nitrogen into a homogenous powder. The pulverized samples (~50 mg) were sonicated for two cycles, 7 s each with a power output of 50J (Model 150 Sonic Dismembrator, Fisher Scientific) in 200 μl of 80 mmol/L Hepes buffer (pH 7.4) containing 300 mmol/L sodium chloride, 20 mmol/L CaCl_2_, 8 mmol/L Triton X‐100, 60% glycerol, 2 mg/ml BSA. The buffer also contained 1 ng prostaglandin E_2_ (PGE_2_)‐d_9_ and 5 ng 5(S)‐hydroxy‐eicosatetraenoc acid (HETE)‐d_8_ as internal standards. To release esterified prostanoids and fatty acid monohydroxides (MHFA) from phospholipids, the samples were incubated for 1 hr at room temperature with soluble phospholipaseA_2_ (sPLA_2_; ~0.9 μmole/min of total activity, Cayman Chemical Co, Ann Arbor, MI). Prostanoids and MHFA were extracted with acetone liquid/liquid extraction by adding 2 ml acetone and 800 μl saline (0.9% NaCl). The samples were centrifuged (2,000*g*; 10 min) and the supernatant was transferred to a new tube. MHFA were extracted from the supernatant using 3 × 2 ml hexane. Following the MHFA extraction, prostanoids were extracted from the same supernatant by acidification of supernatant with formic acid to pH = 3.5 (30 μl of 2 mol/L formic acid), and extraction with 2 ml of chloroform. The chloroform extract containing prostanoids was transferred to glass screw top tubes which were previously silanized with Sigmacote^®^ (Sigma Chemical Co., St. Louis, MO), flushed with nitrogen, and cooled at −80°C for at least 15 min to separate any residual upper phase, which was then removed and discarded before analysis. The extracts were dried down under a stream of nitrogen and transferred to silanized microvial inserts (Micosolv, Eatontown, NJ, USA, part #9502S‐02ND) using 2 × 0.15 ml of either hexane for MHFA or chloroform/methanol (10:1) for prostanoids. The solvent in the microvial was evaporated under a stream of nitrogen, 15 μl methanol/water (1:1) was added, and the samples were vortexed.

#### 2.4.2 UPLC separation and MS analysis

Separation for both the hexane fraction and the chloroform fraction performed on a waters ACUITY UPLC HSS T3 column (1.8 μmol/L, 100 Å pore diameter, 2.1 × 150 mm, Waters, Milford, MA, USA) with an ACUITY UPLC HSS T3 precolumn (1.8 μmol/L, 100 Å pore diameter, 2.1 × 5 mm, waters) at a temperature of 55°C. The liquid chromatography system consisted of a waters ACUITY Class1 UPLC pump with a FTN sampler manager operated at 8°C. Ten μL of sample were injected on a column. The solvent system was composed of 0.1% formic acid in water (solvent A) and 0.1% formic acid in acetonitrile (solvent B). The flow rate was 0.45 ml/min, and the initial solvent conditions were 39% solvent B. The gradient was slightly modified from a previously described method to improve separation of MHFA (Brose, Baker, & Golovko, 2013). The initial conditions were held for 0.5 min, solvent B was increased to 40.5% over 6.88 min, then increased to 70% over 1.62 min, further increased to 75% over 3 min, and finally increased to 98% over 1.5 min. Solvent B was held at 98% for 5.3 min. Solvent B was then returned to the initial conditions over 0.2 min and held for 2 min.

For MS/MS analysis, a triple quadrupole mass spectrometer (Xevo TQ‐S, Waters) with electrospray ionization operated in negative ion mode was used. The capillary voltage was 0.71 kV and the cone voltage was 30 V. The desolvation temperature was 350°C and the source temperature was 150°C. The desolvation gas flow was 1,000 L/hr, the cone gas flow was 150 L/hr, and the nebulizer gas was at 5.0 Bar. MassLynx V4.1 software (Waters) was used for instrument control, acquisition, and sample analysis.

The analytes were monitored in MRM mode using the mass transitions and collision energies presented in Table 2. Prostanoids were quantified using PGE_2_‐d_9_ as an internal standard, while MHFA were quantified using 15‐S‐HETE‐d_8_ as the internal standard.

**Table 2:** Multiple reaction‐monitoring parameters for lipid oxidation products

Analyte	Q1 mass	Q2 mass	Collision energy (V)
PGE_2_	351.2	271.1	16
PGF_2α_	353.1	193.0	20
15Keto‐PGE_2_	349.2	287.1	12
PGE_2_‐d_9_	360.2	280.2	14
9‐HODE	295.1	170.0	16
13‐HODE	295.2	195.0	16
8‐HETE	319.2	155.2	10
9‐HETE	319.2	139.2	10
11‐HETE	319.2	195.2	10
12‐HETE	319.2	179.0	12
15‐HETE	319.2	219.2	10
15‐HETE‐d_8_	327.1	226.1	12
5‐HEPE	317.1	115.0	12
12‐HEPE	317.1	179.0	12
HDOHE	343.1	281.2	12

### 2.5 Statistical analysis

Data (normalized to 100 g of fillet) are reported as mean ± standard deviation. Paired *t* tests were used to compare the fatty acid content of the raw samples to the corresponding cooked samples. *p* values ≤.05 were considered statistically significant. All analyses were done using SAS version 9.4 (SAS Institute, Inc., Cary, NC).

## 3 RESULTS

### 3.1 Fatty acid content

Table 3 presents the fatty acid content of raw and cooked farmed rainbow trout. All of the cooking methods used in this study resulted in no change in the total sum of fatty acids when comparing raw and cooked samples. The general distribution of the fatty acids of the raw samples were PUFA > monounsaturated fatty acids (MUFA) > saturated fatty acids (SFA). The general distribution of fatty acids in the cooked samples varied depending on the cooking method. The predominant SFA, MUFA, and PUFA of both the cooked and raw samples were 16:0, 18:1*n*‐9, and 18:2*n*‐6, respectively.

**Table 3:** Fatty acid content of raw and cooked farmed rainbow trout fillets.^a^^,^ b

Fatty acid	Baked		Broiled		Microwaved	
	Raw	Cooked	Raw	Cooked	Raw	Cooked
SFA						
12:0	4.7 ± 2.2	2.3 ± 2.0	3.8 ± 1.1	1.2 ± 1.1	2.6 ± 2.7	1.0 ± 0.9
14:0	256 ± 82	194 ± 19	215 ± 54	127 ± 32	178 ± 115	119 ± 52
16:0	1,805 ± 510	1,448 ± 109	1,576 ± 319	1,099 ± 244	1,329 ± 772	1,222 ± 394
18:0	515 ± 140	424 ± 34	442 ± 99	302 ± 57	357 ± 195	254 ± 94
20:0	14.7 ± 4.2	12.1 ± 1.2	12.7 ± 2.5	10.6 ± 1.5	11 ± 5.4	8.4 ± 2.8
22:0	10.4 ± 2.4	9.9 ± 3.4	8.2 ± 1.6	6.4 ± 1.0	6.6 ± 4.2	3.9 ± 4.0
24:0	0.7 ± 1.2	0.5 ± 0.9	1.1 ± 1.1	0.9 ± 1.6	0.7 ± 1.2	0.5 ± 0.8
Total SFA	2,606 ± 740	2,091 ± 166	2,259 ± 479	1,547 ± 336	1,885 ± 1,096	1,609 ± 442
MUFA						
18:1*n*‐9	2,506 ± 795	1,928 ± 326	2,160 ± 498	1,365 ± 255	1,791 ± 1,155	1,428 ± 396
20:1*n*‐9	186 ± 55	163 ± 22	163 ± 43	101 ± 14	130 ± 82	83.2 ± 31.6
22:1*n*‐9	20.8 ± 5.5	17.4 ± 1.9	17.8 ± 4.2	11.3 ± 2.3	14.7 ± 10.0	9.6 ± 4.5
24:1*n*‐9	17.8 ± 4.2	16.8 ± 4.0	14.9 ± 3.3	10.4 ± 2.0	11.8 ± 6.1	8.2 ± 3.6
Total MUFA	2,731 ± 859	2,126 ± 355	2,355 ± 547	1,487 ± 273	1,947 ± 1,253	1,529 ± 413
PUFA						
18:3*n*‐3	207 ± 64	159 ± 20	181 ± 39	111 ± 26	154 ± 96	101 ± 37
20:3*n*‐3	20.3 ± 5.7	14.7 ± 1.6	17.2 ± 3.6	10.7 ± 2.4	14.0 ± 8.6	6.9 ± 7.1
20:5*n*‐3	171 ± 45	149 ± 9	148 ± 29	106 ± 19	135 ± 63	72.2 ± 64.7
22:5*n*‐3	84.0 ± 22.7	71.1 ± 6.4	72.9 ± 13.6	49 ± 10.1	67.8 ± 36.5	45.4 ± 15.9
22:6*n*‐3	697 ± 154	667 ± 15	602 ± 117	491 ± 54	546 ± 222	400 ± 122
18:2*n*‐6	1,768 ± 555	1,343 ± 180	1,540 ± 345	1,157 ± 142	1,300 ± 837	1,062 ± 291
18:3*n*‐6	25.2 ± 8.2	20.2 ± 2.6	21.8 ± 5.7	13 ± 1.8	17.6 ± 11.6	10.8 ± 4.5
20:2*n*‐6	149 ± 44	119 ± 15	128 ± 31	79.0 ± 16.8	102 ± 61	53.9 ± 48.2
20:3*n*‐6	75.9 ± 20.2	64.9 ± 5.6	65.0 ± 14.4	45.2 ± 7.5	54.7 ± 27.6	38.6 ± 13.1
20:4*n*‐6	73.8 ± 16.7	75.9 ± 13.7	64.2 ± 11.8	51.4 ± 6.2	55.8 ± 23.3	41.8 ± 13.9
22:2*n*‐6	16.3 ± 4.9	11.5 ± 4.2	13.0 ± 3.8	5.1 ± 2.0	10.3 ± 7.0	2.0 ± 3.5
22:4*n*‐6	16.2 ± 3.5	13.9 ± 0.9	13.2 ± 2.8	9.5 ± 1.1	10.9 ± 6.4	6.8 ± 4.6
EPA + DHA	868 ± 199	816 ± 24	749 ± 145	597 ± 72	680 ± 285	472 ± 183
Total *n*‐3	1,179 ± 292	1,061 ± 48	1,020 ± 201	767 ± 110	916 ± 425	625 ± 242
Total *n*‐6	2,124 ± 652	1,648 ± 219	1,845 ± 414	1,360 ± 174	1,552 ± 973	1,215 ± 307
Total PUFA	3,303 ± 944	2,709 ± 267	2,865 ± 614	2,127 ± 284	2,468 ± 1,398	1,840 ± 455
Total FA	8,639 ± 2,542	6,926 ± 783	7,478 ± 1,636	5,162 ± 890	6,299 ± 3,747	4,978 ± 1,244

Fatty acid	Fried PO		Fried CO		Fried CaO		Fried HOSO	
	Raw	Cooked	Raw	Cooked	Raw	Cooked	Raw	Cooked
SFA								
12:0	1.2 ± 1.3	0 ± 0	2.5 ± 3.0	0.3 ± 0.6	3.4 ± 4.1	0.4 ± 0.7	2.7 ± 2.4	1.0 ± 1.0
14:0	159 ± 27	85.4 ± 13.3^b^	199 ± 105	90.0 ± 17.6	239 ± 156	133 ± 33	178 ± 65	106 ± 57^b^
16:0	1,134 ± 188	849 ± 41	1,451 ± 695	1,108 ± 359	1,709 ± 991	1,067 ± 144	1,241 ± 397	853 ± 392^b^
18:0	312 ± 54	241 ± 12	383 ± 169	253 ± 72	449 ± 232	310 ± 32	330 ± 104	248 ± 101^b^
20:0	10.3 ± 1.9	23.1 ± 3.5	13.0 ± 5.2	18.5 ± 7.0^b^	15.3 ± 6.8	16.9 ± 6.1	11.3 ± 3.4	10.0 ± 3.5
22:0	3.3 ± 2.9	43.4 ± 11.1^b^	6.9 ± 6.6	7.3 ± 4.4	10.8 ± 5.5	11.5 ± 3.8	6.9 ± 3.0	12.4 ± 6.0
24:0	0.2 ± 0.4	21.5 ± 5.9^b^	1.3 ± 1.4	5.0 ± 4.6	0.5 ± 0.8	2.9 ± 2.7	1.0 ± 1.0	4.6 ± 3.0
Total SFA	1,621 ± 268	1,263 ± 49	2,057 ± 985	1,482 ± 464	2,427 ± 1,394	1,541 ± 199	1,772 ± 573	1,235 ± 559^b^
MUFA								
18:1*n*‐9	1,801 ± 297	1,733 ± 94	2,073 ± 1,097	1,745 ± 661	2,526 ± 1,588	2,316 ± 580	1,770 ± 572	1,911 ± 901
20:1*n*‐9	133 ± 28	97.9 ± 8.6	134 ± 65	71.2 ± 18.7	163 ± 92	115 ± 9.7	116 ± 34	81.5 ± 35.5^b^
22:1*n*‐9	14.7 ± 2.7	9.6 ± 1.1	15.8 ± 8.2	6.7 ± 4.6	18.7 ± 10.0	10.9 ± 3.3	14.0 ± 4.1	9.2 ± 4.4^b^
24:1*n*‐9	10.6 ± 1.9	8.0 ± 1.5	11.3 ± 5.7	6.1 ± 5.5	14.7 ± 6.6	11.8 ± 1.5	11.0 ± 2.9	8.6 ± 3.2^b^
Total MUFA	1,959 ± 330	1,848 ± 95	2,234 ± 1,176	1,829 ± 690	2,722 ± 1,696	2,453 ± 572	1,911 ± 613	2,011 ± 940
PUFA								
18:3*n*‐3	153 ± 27	84.1 ± 15.1^b^	189 ± 95	111 ± 31	220 ± 144	145 ± 24	161 ± 55	109 ± 55^b^
20:3*n*‐3	13.8 ± 2.5	7.4 ± 2.1	14.7 ± 8.0	6.4 ± 4.0	17.8 ± 9.6	9.8 ± 4.0	11.7 ± 2.9	9.1 ± 5.4
20:5*n*‐3	130 ± 23	102 ± 9.3	157 ± 62	96.9 ± 21.9	180 ± 91	120 ± 26	137 ± 38	102 ± 36^b^
22:5*n*‐3	72.0 ± 12.4	47.8 ± 5.8	75.9 ± 33.0	43.5 ± 9.2	88.8 ± 47.4	57.2 ± 13.8	67.8 ± 17.5	50.0 ± 17.7^b^
22:6*n*‐3	508 ± 95	456 ± 27	569 ± 171	421 ± 108	650 ± 231	497 ± 81	505 ± 104	409 ± 90^b^
18:2*n*‐6	1,294 ± 218	981 ± 62	1,597 ± 845	2,392 ± 1,105	1,861 ± 1,262	1,138 ± 230	1,361 ± 465	952 ± 486^b^
18:3*n*‐6	17.5 ± 2.9	9.8 ± 2.0	20.7 ± 10.5	10.3 ± 3.0	24.2 ± 15.5	14.6 ± 2.7	17.7 ± 5.3	11.5 ± 5.7^b^
20:2*n*‐6	106 ± 19	60.2 ± 10.8^b^	109 ± 51	54.1 ± 12.9	130 ± 73	78.7 ± 18.0	96.0 ± 24.9	65.3 ± 29.0^b^
20:3*n*‐6	41.1 ± 7.0	27.6 ± 3.7^b^	45.9 ± 19.0	26.5 ± 5.2	58.0 ± 25.6	38.6 ± 7.9	40.1 ± 9.8	28.8 ± 11.3^b^
20:4*n*‐6	54.6 ± 8.7	47.0 ± 2.5	65.7 ± 19.9	43.8 ± 10.1	72.5 ± 30.4	53.3 ± 10.3	57.3 ± 13.8	43.8 ± 11.8^b^
22:2*n*‐6	5.7 ± 5.0	0 ± 0	9.7 ± 7.6	1.2 ± 2.1	9.9 ± 10.3	3.4 ± 3.7	6.9 ± 2.1	5.7 ± 3.9
22:4*n*‐6	11.5 ± 1.7	8.3 ± 2.1	11.6 ± 6.7	6.8 ± 5.9	15.3 ± 7.3	8.3 ± 5.3	12.1 ± 2.9	8.8 ± 3.2^b^
EPA + DHA	638 ± 118	558 ± 35	725 ± 232	518 ± 130	830 ± 322	617 ± 107	642 ± 142	511 ± 126^b^
Total *n*‐3	877 ± 160	697 ± 52	1,005 ± 368	679 ± 173	1,156 ± 522	829 ± 145	882 ± 216	679 ± 204^b^
Total *n*‐6	1,530 ± 257	1,133 ± 77	1,859 ± 959	2,534 ± 1,141	2,171 ± 1,421	1,335 ± 260	1,591 ± 522	1,116 ± 551^b^
Total PUFA	2,406 ± 417	1,830 ± 113	2,864 ± 1,326	3,213 ± 1,313	3,327 ± 1,944	2,164 ± 405	2,473 ± 737	1,795 ± 755^b^
Total FA	5,986 ± 1,014	4,942 ± 186	7,155 ± 3,484	6,523 ± 2,466	8,475 ± 5,025	6,158 ± 735	6,156 ± 1,922	5,040 ± 2,202

a Data expressed as mg/100 g fish (wet weight) and represented as mean ± SD, *n* = 3.

b *p* ≤ .05.

### 3.2 Total fatty acids

Microwaving, baking, and oven broiling had no significant effect on the total fatty acid content of the fillets. Pan‐frying had different effects on the fatty acid content depending on the type of oil used during cooking. Pan‐frying in CO, CaO, or PO did not alter the total fatty acid content of the fillets. When the fillets were fried in HOSO, the amount of total SFA, total *n*‐3 fatty acids, total *n*‐6 fatty acids, and total EPA + DHA were reduced.

### 3.3 Saturated fatty acids

SFA content was not altered when the samples were pan‐fried in CaO. Pan‐frying in PO reduced 14:0 and increased 22:0 and 24:0. When the fillet was pan‐fried in CO, 20:0 was increased. Pan‐frying in HOSO reduced 14:0, 16:0, and 18:0.

### 3.4 Monounsaturated fatty acids

MUFA content was not altered when the fillets were pan‐fried in PO, CO, or CaO. Pan‐frying in HOSO reduced 20:1*n*‐9, 22:1*n*‐9, and 24:1*n*‐9 relative to the raw fillet, though they were relatively minor species.

### 3.5 Polyunsaturated fatty acids

PUFA content was not altered when samples were pan‐fried in CO or CaO. Pan‐frying in PO decreased 18:3*n*‐3, 20:2*n*‐6, and 20:3*n*‐6 levels. When pan‐fried in HOSO, there was a reduction in 18:3*n*‐3, 20:5*n*‐3, 22:5*n*‐3, 22:6*n*‐3, 18:2*n*‐6, 18:3*n*‐6, 20:2*n*‐6, 20:3*n*‐6, 20:4*n*‐6, and 22:4*n*‐6 compared to the raw fillets.

### 3.6 Oxylipin content

**Figure 1:**
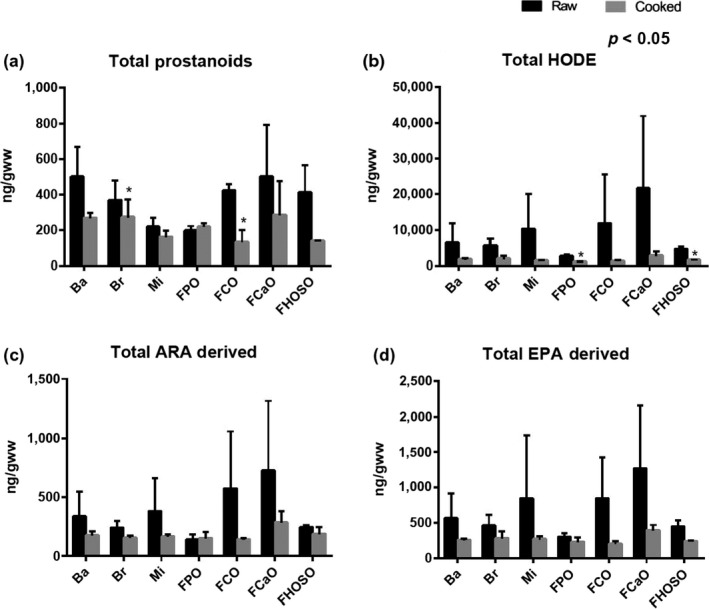
Changes in oxylipin content of fillets cooked by various methods compared to raw fillets. Data expressed as ng/g of wet weight (gww) and represented as mean ± *SD*. *p ≤ .05. Black columns represent raw samples, gray columns represent cooked samples. Ba, baking; Br, broiling; FCaO, pan‐fried in canola oil; FCO, pan‐fried in corn oil; FPO, pan‐fried in peanut oil; FHOSO, pan‐fried in high oleic sunflower oil; Mi, microwaving; PO, peanut oil

The effect of cooking on total oxylipin content of the fillets was dependent on both the cooking method and oxylipin under examination (Figure 1). Microwaving, baking, and pan‐frying in CaO had no significant effect on the total oxylipin content of the fillets. Oven broiling and pan‐frying in CO decreased total prostanoid levels of the cooked fillet. Pan‐frying in PO or HOSO decreased total hydroxy‐octadecadienoic acid (HODE) levels of cooked fillet.

EPA‐ and DHA‐derived oxylipins were not altered, while various arachidonic acid (ARA; 20:4*n*‐6) and linoleic acid (LA; 18:2*n*‐6)‐derived oxylipins were reduced with different cooking methods (Table 4). Microwaving and baking decreased the ARA‐derived oxylipin prostaglandin *F*_2α_ (PGF_2α_), while oven broiling resulted in a decrease of PGE_2_ and PGF_2α_. When the fillets were fried in CaO, there was no significant difference in oxylipin content relative to the raw fillets. Pan‐frying in CO resulted in a decrease of PGE_2_ and PGF_2α_. When pan‐fried in PO, PGF_2α_, 15‐keto‐PGE_2_, and 13‐HODE. Frying in HOSO resulted in a decrease of oxylipins 11‐HETE, 9‐HODE, and 13‐HODE.

**Table 4:** Oxylipin content of raw and cooked farmed rainbow trout.^a^^,^ b

Oxylipin	Parent FA	Baked		Broiled		Microwaved	
		Raw	Cooked	Raw	Cooked	Raw	Cooked
PGF_2α_	ARA	18.4 ± 3.3	3.6 ± 0.7^b^	16.6 ± 4.5	3.5 ± 1.8^b^	10.6 ± 3.0	1.3 ± 1.0^b^
PGE_2_	ARA	476 ± 166	265 ± 27	348 ± 107	270 ± 94^b^	205 ± 50	160 ± 38
15‐keto‐PGE_2_	ARA	5.2 ± 3.6	0.5 ± 0.3	2.6 ± 1.4	0.2 ± 0.1	2.5 ± 1.7	0.7 ± 0.5
8‐HETE	ARA	20.3 ± 14.1	8.1 ± 0.3	14.5 ± 1.7	8.5 ± 1.8	23.6 ± 16.9	7.2 ± 1.0
9‐HETE	ARA	0.8 ± 0.6	0.3 ± 0.2	0.5 ± 0.2	0.5 ± 0.1	0.7 ± 0.6	0.5 ± 0.2
11‐HETE	ARA	2.8 ± 1.7	1.5 ± 0.5	2.0 ± 0.6	1.2 ± 0.4	3.3 ± 2.6	1.1 ± 0.3
12‐HETE	ARA	128 ± 63	60.4 ± 2.7	112 ± 23	73.7 ± 10.8	157 ± 90	69.4 ± 4.6
15‐HETE	ARA	185 ± 136	105 ± 36	109 ± 41	71.2 ± 15.8	195 ± 177	85.5 ± 18.2
9‐HODE	LA	2,893 ± 2,546	733 ± 161	2,456 ± 913	759 ± 352	4,684 ± 4,526	58 ± 42
13‐HODE	LA	3,521 ± 2,959	962 ± 242	3,076 ± 1,091	1,082 ± 565	5,587 ± 5,234	803 ± 63
5‐HEPE	EPA	281 ± 188	142 ± 22	203 ± 60	114 ± 44	355 ± 369	115 ± 20
12‐HEPE	EPA	281 ± 165	113 ± 5	258 ± 92	168 ± 53	491 ± 523	151 ± 28
HDOHE	DHA	2,498 ± 1,671	1,716 ± 56	1,737 ± 461	1,589 ± 455	3,156 ± 3,019	1,248 ± 397

Oxylipin	Parent FA	Fried PO		Fried CO		Fried CaO		Fried HOSO	
		Raw	Cooked	Raw	Cooked	Raw	Cooked	Raw	Cooked
PGF_2α_	ARA	14.9 ± 2.5	2.8 ± 0.5^b^	12.8 ± 1.3	2.9 ± 1.1^b^	11.0 ± 1.5	6.0 ± 5.3	13.6 ± 5.4	2.0 ± 0.5
PGE_2_	ARA	181 ± 26	216 ± 21	406 ± 36	131 ± 67^b^	482 ± 282	277 ± 185	394 ± 155	136 ± 2
15‐keto‐PGE_2_	ARA	1.2 ± 0.5	0.2 ± 0.2^b^	1.8 ± 0.5	0.3 ± 0.2	7.5 ± 10.1	1.3 ± 1.1	4.2 ± 3.5	0.8 ± 1.0
8‐HETE	ARA	7.7 ± 2.9	7.9 ± 3.3	32.6 ± 28.2	7.3 ± 1.3	47.5 ± 42.9	12.3 ± 4.0	13.7 ± 0.6	8.8 ± 3.2
9‐HETE	ARA	0.3 ± 0.2	0.1 ± 0.0	1.3 ± 1.0	0.1 ± 0.1	3.2 ± 4.2	0.3 ± 0.2	0.5 ± 0.1	0.2 ± 0.1
11‐HETE	ARA	1.3 ± 0.2	1.0 ± 0.6	5.2 ± 5.1	0.8 ± 0.2	5.9 ± 5.0	2.0 ± 1.0	2.0 ± 0.3	1.0 ± 0.2^b^
12‐HETE	ARA	82.9 ± 25.6	89.8 ± 35.1	257 ± 151	71.4 ± 19.3	280 ± 178	144 ± 78	131 ± 17	89.5 ± 35.2
15‐HETE	ARA	49.7 ± 13.6	53.4 ± 14.0	277 ± 302	59.7 ± 7.6	387 ± 367	125 ± 21	98.1 ± 2.0	88.2 ± 25.9
9‐HODE	LA	1,108 ± 266	415 ± 62	5,249 ± 6,110	527 ± 95	9,587 ± 9,113	1,098 ± 443	2,002 ± 387	624 ± 8^b^
13‐HODE	LA	1,452 ± 258	611 ± 78^b^	6,602 ± 7,607	760 ± 112	12,005 ± 11,233	1,635 ± 764	2,539 ± 446	890 ± 101^b^
5‐HEPE	EPA	117 ± 23	71 ± 26	410 ± 350	76.7 ± 3.2	603 ± 477	172 ± 22	172 ± 35	107 ± 23
12‐HEPE	EPA	181 ± 31	160 ± 45	435 ± 235	124 ± 40	665 ± 417	222 ± 82	273 ± 60	131 ± 12
HDOHE	DHA	1,007 ± 143	1,202 ± 455	3,248 ± 2,393	1,053 ± 157	4,109 ± 2,646	1,974 ± 544	1,659 ± 241	1,536 ± 425

a Data expressed as ng/gww and represented as mean ± SD, *n* = 3.

b *p* ≤ .05.

## 4 DISCUSSION

We hypothesized that cooking method would have no effect on the fatty acid content of the fillets. From this study, we found that pan‐frying was the only cooking method that impacted the fatty acid content. Previous studies evaluating the effect of cooking methods on the fatty acid profile of various fish species have also found that pan‐frying has the largest impact (Sioen et al., 2006). Pan‐frying created the largest changes in fatty acid content likely due to the fatty acid composition of the culinary oils. The effect the culinary fat has on the fatty acid distribution of the fish fillet depends on the type of fish and the fatty acid profile of the culinary fat used (Nieva‐Echevarría, Goicoechea, Manzanos, & Guillén, 2016; Sioen et al., 2006). The culinary fat being used will also be altered as lipids are transferred from the fillet to the oil (Sioen et al., 2006). Exchange of lipids also occur the other direction (oil to fillet), but not to the same extent. Studies have noted that when pan‐frying fatty fish, the predominant fatty acid of the oil actually increases in the fillet (Al‐Saghir et al., 2004; Sioen et al., 2006). Our results follow this trend. Differences in our results compared to other studies may be due to variation in cooking method and the fatty acid content of the fillets. Even so, the exchange of fatty acids between the fillet and the culinary oil would explain some of the changes in fatty acid content observed in the current study.

We observed that pan‐frying in HOSO significantly decreased total *n*‐3 PUFA, total *n*‐6 PUFA, EPA, DHA, and total SFA levels. These results were similarly observed in a study conducted by Ansorena et al. (2010) on salmon which showed that pan‐frying in SO significantly changed the lipid profile even though the total fat content was unchanged. They found the amount of total SFA, MUFA, and PUFA were significantly lower in the fried sample than the raw sample. In addition, the individual fatty acid content was significantly altered. There were slight, but significant, decreases in the levels of EPA, DHA, 16:0, and 18:3*n*‐3, which our study also observed.

Pan‐frying in CO resulted in minimal changes in the fatty acid profile. Another study observed similar results with no changes in SFA, MUFA, PUFA, EPA, and DHA levels when salmon was fried in CO (Al‐Saghir et al., 2004). These observations likely resulted due to the composition of the fish fillet and of the oil. As illustrated in Table 1, CO has a general FA distribution of PUFA > MUFA > SFA with the predominant FA being 18:2*n*‐6 (Hosseini, Ghorbani, Meshginfar, & Mahoonak, 2016). Both farmed rainbow trout and farmed salmon contain a high level of PUFA (Weaver et al., 2008). Therefore, there would likely be minimal movement of fatty acids. Our study, like previous studies, showed no significant change in the fatty acid composition of the fillet when pan‐fried in CO.

When pan‐fried in CaO no differences were found in the fatty acid profile of the fried compared to the raw fillet. Agren and Hanninen (1993) found that pan‐frying rainbow trout in CaO resulted in small increases of 18:1*n*‐9, 18:2*n*‐6, and 18:3*n*‐3. These results differ from ours because the skin of the trout was kept on during the cooking process in the other study which is thought to prevent the transfer of lipids between the fillet and the culinary oil, but still allowed the loss of moisture (Agren & Hanninen, 1993).

To our knowledge, this is the first study to observe the change in fatty acid content when pan‐frying fish in PO. As illustrated in Table 1, the predominant fatty acids in PO are 18:1*n*‐9 and 18:2*n*‐6 (Hosseini et al., 2016). The movement of fatty acids from the fillet to the oil would favor the movement of PUFA. The significant decrease of 18:3*n*‐3 in the fillet was likely due to the movement of this fatty acid to the culinary oil.

We hypothesized that cooking would increase lipid oxidation and thus the content of oxylipins due to the exposure to high temperatures. We observed decreases in the content of some oxylipins of cooked versus raw fillets, depending on the cooking technique. Our previous work found similar results after baking salmon (Raatz et al., 2011). In addition, other studies found that pan‐frying had minimal thermal oxidation of the lipids in the fillet (Al‐Saghir et al., 2004; Nieva‐Echevarría et al., 2016). The decrease in oxylipins content after cooking therefore was likely due to decrease in fatty acid and preexisting oxylipins content, as the fillets were minimally exposed to thermal oxidation.

Baking, oven broiling, microwaving, and pan‐frying in CO decreased the prostanoid content, the likely result of decomposition of the prostanoids. As oxylipins are derived from fatty acids, the observation of no change in oxylipin content when pan‐frying in CaO reflects the unaltered fatty acid content. Pan‐frying in PO resulted in a decrease in prostanoids and 13‐HODE. This was likely due to decomposition of the prostanoids and perhaps as a result of decreased *n*‐6 PUFA in the fillet.

The change in fatty acid content when pan‐frying in HOSO complements the changes in oxylipin content. Total HODE (9‐HODE, 13‐HODE) levels decreased likely due to the decrease in 18:2*n*‐6 in the fillet as these oxylipins are derived from this fatty acid. The decrease in 20:4*n*‐6 likely explains the observed decrease in 11‐HETE. The other eicosanoids decreased, but not significantly due to the large standard deviations.

This study provides new insight into how the fatty acid and oxylipin composition of rainbow trout is impacted by various cooking methods. There remain, however, a few limitations to this study. A primary limitation is the large standard deviations of our results. This can be rectified in the future by analyzing a greater number of fillet samples. Another reason for the large standard deviations could be due to real variation in the fatty acid content of the fillets used. Across fillet variation was controlled as much as possible by using fish hatched and reared in the same environment and cutting samples from similar parts of the fillets. Even so, there still could have been within fillet variation in the composition of the fish due to the location of where the samples were taken from each of the fillets (Fjellanger, Obach, & Rosenlund, 2001; Testi, Bonaldo, Gatta, & Badiani, 2006). Another limitation of this study was that the culinary oils used were not analyzed after the fish was prepared; therefore, the true movement of the fatty acids was unknown. Previous research has shown that there is an interaction between the fatty acids of the fillet and the culinary fat used (Nieva‐Echevarría et al., 2016; Sioen et al., 2006). Even with these limitations, this study is novel because it not only observed the changes in fatty acid composition of farmed rainbow trout, but also the oxylipin content.

Future research is needed with a larger sample size in order to confirm the results of this study. In addition, research is needed to determine the health impact of oxylipin compounds in the fillet.

## 5 CONCLUSIONS

In summary, pan‐frying was the only cooking method that resulted in a significant change in the overall fatty acid profile of the farmed rainbow trout. Changes in the oxylipin content varied by cooking method, however, the physiological impact of these changes is currently unknown. Therefore, cooking methods of rainbow trout that would optimize the consumption of *n*‐3 PUFA are baking, broiling, microwaving, or pan‐frying in CO, CaO, or PO as these cooking methods did not significantly impact the fatty acid profile of the fillets.

## CONFLICT OF INTEREST

The authors declare no conflict of interest.
